# Multi‐Targeted Kinase Inhibitor Therapy in Pediatric Bone and Soft Tissue Sarcoma Patients—A Single Centre Experience

**DOI:** 10.1002/cam4.70951

**Published:** 2025-05-09

**Authors:** Anna Mohás, Klára Horváth, Zsuzsanna Jakab, Monika Csóka

**Affiliations:** ^1^ Pediatric Centre, Tűzoltó Street Department Semmelweis University Budapest Hungary; ^2^ National Childhood Cancer Registry (NCCR), Hungarian Pediatric Oncology Network (HuPON). Department of Paediatrics Semmelweis University Budapest Hungary

**Keywords:** osteosarcoma, pazopanib, pediatric, regorafenib, soft tissue sarcoma, sorafenib

## Abstract

**Background:**

Patients with relapsed or refractory soft tissue and bone sarcomas have dismal outcomes. Multi‐targeted kinase inhibitors (mTKI) have proven to be potent agents in several malignancies, both as primer therapy and as a salvage option. Our aim was to evaluate the clinical outcomes of mTKI treatment in a heterogeneous group of pediatric sarcoma patients retrospectively.

**Procedures:**

A total of 18 patients were treated with sorafenib, regorafenib, or pazopanib; 13 of them had osteosarcoma (OSC), 3 had synovial sarcoma (SySa), and 1–1 patient had chondrosarcoma and rhabdomyosarcoma. Indication for mTKI treatment was primarily progressive, inoperable, relapsed, or chemotherapy‐resistant disease after completion of first‐ and second‐line chemotherapy.

**Results:**

At the time of the beginning of mTKI treatment, the median age was 16.5 years, and the median time to progression from initiation of mTKI was 4 months. The overall response rate was 16%. We conducted a comparison of the survival outcomes of OSC patients receiving mTKIs against a retrospective, non‐randomized control group. Overall survival was evaluated from the time of progression or relapse after second‐line treatment to the time of death. The log‐rank test revealed a significant difference in the survival distribution between patients receiving mTKIs and those who did not (chi2(1) = 8.13 *p* = 0.004). We observed benefits from mTKI treatment in 3 SySa patients, with pazopanib demonstrating effectiveness and no progression observed thus far.

**Conclusions:**

Our findings suggest that mTKIs are well‐tolerated and can serve as a therapeutic option for refractory bone sarcomas as palliative treatment, aiming to slow disease progression and uphold a good quality of life.

**Abbreviations**
AE: adverse effect
CR: complete remission
CSC: chondrosarcoma
FGFR: fibroblast growth factor receptor
IQR: interquartile range
MAP/ERK: mitogen‐activated protein kinase/extracellular signal regulated kinase
mTKI: multi‐targeted kinase inhibitor
OSC: osteosarcoma
PDGFR: platelet‐derived growth factor receptor
PFS: progression free survival
RMS: rhabdomyosarcoma
STS: soft tissue sarcoma
SySa: synovial sarcoma
VEGF: vascular endothelial growth factor

## 1 Introduction

Approximately 230–250 children are diagnosed with malignant diseases yearly in Hungary. Among them, 2% have osteosarcoma (OSC) and 5.4% have soft tissue sarcomas (STS). OSC is treated with multi‐agent chemotherapy and radical surgical resection of the tumor. With this multidisciplinary approach, up to 70% of patients with localized disease can become long‐term survivors. The prognosis is poorer in cases of progressive, relapsed, and primarily metastatic disease. The overall survival in these cases was reported to be around 30% [1]. Soft tissue sarcomas are a heterogenous group of diseases, with rhabdomyosarcomas (RMS) being most common among children. Despite the good prognosis of localized RMS, the survival for relapsed patients remains poor [2]. The remaining diseases, collectively referred to as nonrhabdomyosarcoma soft‐tissue sarcomas (NRSTS), comprise a diverse group of neoplasms. Over 140 distinct types of NRSTS have been identified, with notable variations in incidence across different age groups. In children, the most common variants are infantile fibrosarcoma and malignant rhabdoid tumor (MRT), while in adolescents, synovial sarcoma (SySa) and malignant peripheral nerve sheath tumor (MPNST) occur more frequently [3]. These tumors exhibit a broad spectrum of biological behavior and require tailored treatment approaches, including varying systemic chemotherapy regimens, highlighting the importance of an accurate pathological diagnosis for appropriate management.

Multi‐targeted kinase inhibitors (mTKIs) have proven to be potent agents in several malignancies, both as primer therapy and as a salvage option. mTKIs have a broad inhibition profile, which has been shown to target tumor angiogenesis. Many mTKIs are under investigation and used in clinical practice. Sorafenib is an anticancer drug that works by inhibiting the mitogen‐activated protein kinase/extracellular signal regulated kinase (MAP/ERK) pathway [4]. It was first approved to treat hepatocellular carcinoma and renal cell carcinoma. Sorafenib has also demonstrated activity as a second‐ or third‐line treatment in osteosarcoma, both as monotherapy and in combination. In a non‐randomized phase II study involving patients over 14 years old with metastatic, relapsed, and unresectable osteosarcoma, sorafenib showed efficacy, with a progression‐free survival (PFS) rate of 46% at 4 months [5, 6].

Regorafenib is an mTKI that blocks the activity of various protein kinases involved in angiogenesis (VEGF 1, 2 and 3) and oncogenesis (KIT, RET, RAF‐1 and BRAF). Regorafenib shows significant activity in refractory colorectal carcinoma [7]. It was also found effective in metastatic osteosarcoma in a double‐blind phase II study, where median PFS was significantly improved with regorafenib versus placebo: 3.6 months versus 1.7 months, respectively [8].

Pazopanib exerts its antitumor effect by inhibiting vascular endothelial growth factor (VEGF)‐ mediated angiogenesis and by directly blocking growth‐promoting receptor tyrosine kinases such as platelet‐derived growth factor receptors (PDGFRs) and fibroblast growth factor receptors (FGFRs). It was first authorized to treat metastatic renal cell carcinoma and became the first mTKI licensed for the treatment of multiple types of STS based on the results of a phase III trial. This trial (PAzopanib expLorEd in SofT‐Tisue Sarcoma—a phasE 3 study; PALETTE) was designed to compare the efficacy of pazopanib with placebo for metastatic, progressive soft‐tissue sarcoma, and it resulted in a median progression‐free survival of 4.6 months for pazopanib compared with 1.6 months for placebo. Overall survival was 12.5 months with pazopanib versus 10.7 months with placebo [9].

The promising results with the above mentioned mTKIs, coupled with their moderate toxicity profiles, suggest that sorafenib, regorafenib, and pazopanib could be considered as treatment options for young patients with refractory OSC and STS. The studies conducted with the aforementioned drugs were predominantly performed in adult populations. Therefore, we found it important to summarize our practical experiences and real‐world data gathered in children.

Our aim was to evaluate the clinical outcomes of mTKI treatment in a heterogeneous group of pediatric sarcoma patients, focusing on survival rates, toxicities, and overall quality of life.

## 2 Materials and Methods

### 2.1 Patients

All patients diagnosed with OSC, STS, and chondrosarcoma (CSC) at the Pediatric Centre, Tűzoltó street Department, Semmelweis University (Budapest, Hungary) during the period 2013–2023 were retrospectively reviewed. The data of patients treated with sorafenib, regorafenib, or pazopanib were analyzed. OSC treatment was based on the EURAMOS protocol (cisplatin, doxorubicin, high dose methotrexate) [1], chondrosarcoma treatment followed the COSS‐96 protocol (cisplatin, doxorubicin, high dose methotrexate, ifosfamide) [10] and the STS patients were treated according to the CWS‐2012 protocol (vincristine, ifosfamide, actinomycin‐D, carboplatin, etoposide, doxorubicin) [11]. Given that OSC patients represented the largest cohort, we conducted a comparative analysis involving this group.

The study was approved by the Ethics and Scientific Committee of Semmelweis University (project identification code: 99/2018). Written informed consent of the participants' legal guardian or next of kin was not required to participate in this study in accordance with the national legislation and the institutional requirements.

### 2.2 Treatment

During this period, indication of mTKI treatment was based on the clinician's and patient's decision, in cases when all other conventional therapeutic approaches failed.

Sorafenib was administered at a dose of 400 mg twice a day for patients weighing more than 50 kg. For younger patients, the dose was calculated based on their body surface area, with a dose of 400 mg/m^2^.

Regorafenib was given at a dose of 82 mg/m^2^, with a maximum dose of 160 mg daily during the first 21 days of each 28‐day cycle.

Pazopanib was administered at a dose of 350–450 mg/m^2^, with a maximum dose of 800 mg/day. Treatment was continued until disease progression or unbearable toxicity.

### 2.3 Assessment of Response and Toxicity

All patients had regular tumor imaging using chest CT, MRI of the affected limb if no implants were inserted, or bone scintigraphy at the beginning of the treatment and every 3 months thereafter. The type of radiological method was chosen according to the location of the tumor and/or metastases. During the monthly visits, a physical examination and laboratory tests (complete blood count, electrolytes, liver and kidney function) were performed. Data on mortality were obtained through the electronic medical record system. The Karnofsky‐Lansky score and all toxicities were documented until the end of the therapy. mRECIST criteria were used to evaluate the response [12].

### 2.4 Statistical Analyses

Descriptive statistics of patients and treatments are presented as medians and interquartile ranges (IQR) due to non‐normal distribution. Summary statistics are also provided separately for the largest disease group, OSC patients. Baseline characteristics were compared using independent t‐tests for continuous variables and Fisher's exact test for binary variables. In addition, survival analysis was conducted to compare OSC patients receiving mTKI to controls. The log‐rank test and Cox regression with age, metastatic disease, and initial therapeutic response based on the post‐operative histological results as covariates were performed. The assumptions of Cox regression were assessed graphically and with the test of proportional hazard assumption. STATA/BE 18.0 (StataCorp. 2023. Stata Statistical Software: Release 18 College Station, TX: StataCorp LLC) was utilized for all statistical analyses.

## 3 Results

### 3.1 Description of Patients Receiving mTKI

**FIGURE 1:**
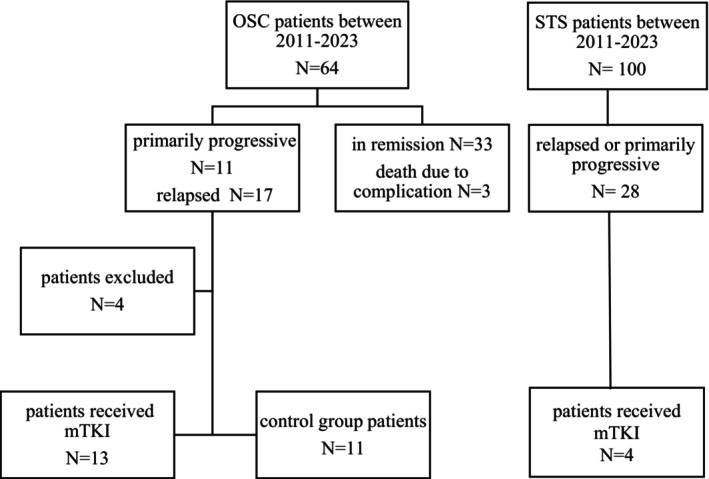
Study inclusion criteria flow chart. A total of 64 patients were diagnosed with OSC between 2011 and 2023 in the 2nd Department of Pediatrics, Semmelweis University. A total of 33 patients are in remission, 3 patients died due to treatment complications. A total of 11 patients had primarily progressive disease, and 17 patients had relapsed disease. We excluded 3 patients due to incomplete first‐line treatment. One patient was excluded because of a good therapeutic response to second‐line treatment. A total of 13 patients received mTKI. The remaining relapsed or primarily progressive patients, who showed poor response to first‐ and second‐line therapy, were included in the control group. A total of 100 patients were diagnosed with STS in this period. A total of 28 of them had primarily progressive or relapsed disease. A total of 4 received mTKI. Given the low number of cases, we did not conduct a study with a control group in the STS cohort.

Between 2013 and 2023, 64 patients were diagnosed with OSC and 100 patients with STS in our institution (Figure 1). A total of 18 patients received treatment with oral mTKIs. Of these, 13 had OSC, three had SySa, one had RMS, and one had mesenchymal chondrosarcoma (CSC). The patients' clinical characteristics are shown in Table 1. The median age at the time of diagnosis was 14.8 years (range: 5.9–17.6, IQR: 10.7–16.1), while the median age at the initiation of mTKI therapy was 16.5 years (range: 8.5–18.7, IQR: 16.5–8.5). The median time to 1st relapse or progression when second‐line chemotherapy was initiated was 15.5 months (range: 2–75, IQR: 5.75–20.25), and the median time from initial diagnosis to the start of mTKI treatment was 20 months (range: 3–85, IQR: 9.5–31). The median time to progression from initiation of mTKI was 4 months (range: 1–16, IQR: 10.3–16.15). The median follow‐up period from the start of mTKI was 7.5 months (range: 1–32, IQR: 3–14.2). At the time of diagnosis, 12 patients had metastatic disease. Karnofsky‐Lansky score was recorded at each follow‐up visit throughout the mTKI treatment. The median score after 1 month of treatment was 80 (range: 20–100 IQR: 47.5–92.5).

**FIGURE 2:**
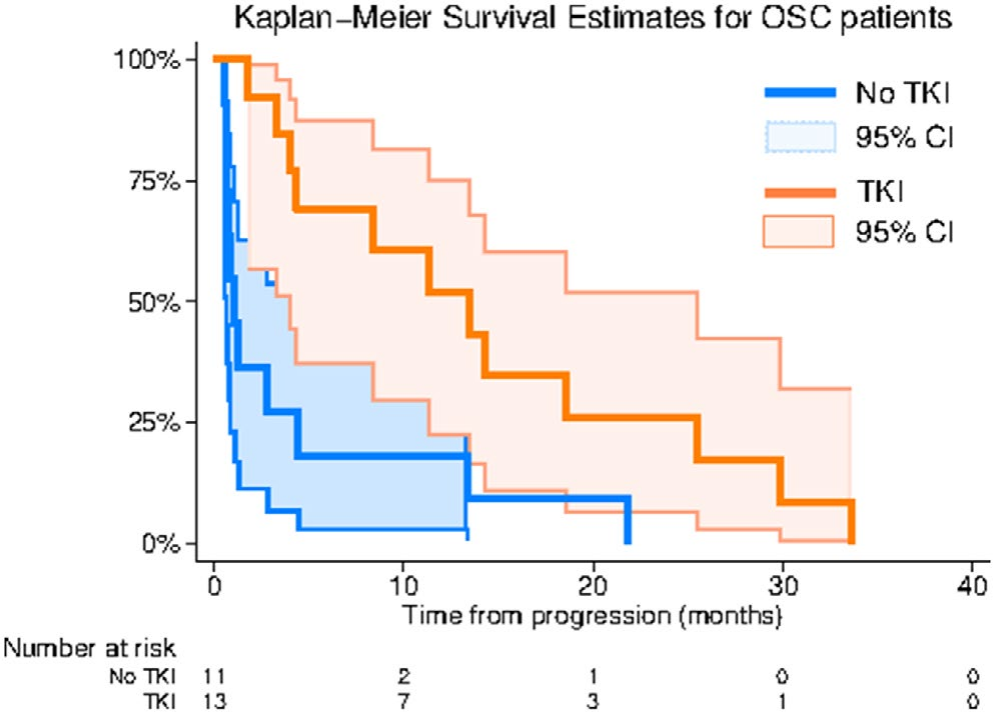
Kaplan–Meier survival curves with 95% confidence intervals for OSC patients receiving mTKI or not.

**TABLE 1:** Patient's characteristics.

No.	Diagnosis	Sex (male/female)	Age at diagnosis (years)	Age at mTKI treatment (years)	Status at diagnosis (localized/metastatic)	Type of mTKI (sorafenib/regorafenib/pazopanib)	Time to relapse/progression from diagnosis (month)	Time to progression from mTKI (month)	Length of therapy (months)	Best response	Karnofsky‐Lansky score after 1 months	Current status
1	Osc	m	15	16.8	loc	S	20	3	3	PD	60	DOD
2	Osc	m	5.9	9.3	met	S	19	14	28	SD	90	DOD
3	Osc	m	6.9	8.6	met	S	16	8	13	SD	90	DOD
4	Osc	m	15.3	18.7	loc	P, R	21	16	18	SD	70	DOD
5	Osc	m	16.1	17.9	met	S	15	1	1	PD	40	DOD
6	Osc	m	17	18.1	met	S, R, P	16	16	24	SD	80	DOD
7	Osc	m	17.6	18.5	met	S, R, P	10	9	32	SD	100	DOD
8	Osc	m	15.4	16.2	met	P	6	2	2	PD	20	DOD
9	Osc	m	13.2	16.8	met	S	23	1	1	PD	60	DOD
10	Osc	f	10.7	12.5	met	S, R	17	3	12	PD	50	DOD
11	Osc	f	14.6	16.4	met	R	15	4	Under therapy	SD	90	AWD
12	Osc	m	16.9	17.4	met	R	5	3	3	SD	40	DOD
13	Osc	m	13.1	14	met	S	7	5	8	PD	80	DOD
14	Csc	f	13.8	16.1	met	P	25	11	6	SD	80	DOD
15	Rms	m	15.9	17.1	loc	R	3	1	5	PD	40	DOD
16	SySa	f	16.3	16.6	loc	P	2	No prog.	11	CR	100	NED
17	SySa	m	9	9.5	loc	P	3	No prog.	Under therapy	PR	100	AWD
18	SySa	m	9.4	16.5	loc	P	75	No prog.	Under therapy	PR	100	NED

Abbreviations: AWD, alive with disease; CR, complete response; CSC, chondrosarcoma; DOD, died of disease; loc, localized disease; met, metastatic disease; NED, no evidence of disease; OSC, osteosarcoma; PD, progressive disease; PR, partial response; RMS, rhabdomyosarcoma; SD, stable disease; SySa, synovial sarcoma.

### 3.2 Outcome for Osteosarcoma Patients

The indication for using mTKIs in OSC was for relapsed, chemotherapy‐resistant disease. It was administered as palliative treatment after multiple chemotherapy combination regimens proved ineffective. All patients initially received first‐line chemotherapy based on methotrexate, adriamycin, and cisplatin. As a second‐line chemotherapy, carboplatin, ifosfamide, and etoposide were given, with two patients also receiving gemcitabine and docetaxel due to resistance to other agents. Surgical resection of primary and metastatic lesions was performed in operable cases, while palliative radiotherapy was administered in inoperable cases.

mTKI therapy was initiated after the first relapse or primary progression in 8 cases, after the second relapse in 4 cases, and after the third relapse in 1 case. The median duration of mTKI therapy was 10 months (range: 1–32, IQR: 3–13). Stable disease was achieved in 7 cases (53%), while disease progression was observed in 6 cases (47%) according to mRECIST criteria. The median time to disease progression was 4 months (range: 1–16, IQR: 2.25–12.75). Sorafenib was the primary treatment choice in 5 cases, regorafenib in 3, and pazopanib in 2 cases. In addition, 3 patients received multiple types of mTKIs. Presently, one patient is alive with progressive disease and is undergoing regorafenib therapy. The median time from the start of mTKI to death was 9.5 months (range: 1–32, IQR: 2.25–22.25).

### 3.3 Comparison With Control Group

A total of 64 patients were diagnosed with OSC at the Pediatric Centre, Tűzoltó street Department, Semmelweis University between 2011 and 2023. Among them, 28 patients had primarily progressive or relapsed disease. We performed a comparison of the survival outcomes between OSC patients receiving mTKIs and a control group. Inclusion criteria were age 0–18 years; primarily progressive, inoperable, relapsed, or chemotherapy‐resistant disease; and completion of first‐line chemotherapy. Exclusion criteria were incomplete first‐line treatment and good therapeutic response for second‐line treatment. Four patients were excluded either due to incomplete first‐line treatment (*N* = 3) or showing a good therapeutic response to second‐line treatment (*N* = 1). Patients receiving mTKI were assigned to the treatment group, while all others were considered controls. The participant flow chart is presented in Figure 1.

No significant differences were observed between the treatment and control groups regarding age distribution, sex, metastases, and chemotherapeutic response. Detailed results can be found in Table 2. Both the treatment and control groups underwent primary and secondary treatment based on the same chemotherapy regimen.

**TABLE 2:** Baseline study characteristics.

	mTKI (*N* = 13)	Control (*N* = 11)	*p*
Age at diagnosis (years), mean and SD	13.6 (±3.73)	13.3 (±3.17)	0.718
Male, *n*	11	8	0.630
Metastatic disease, *n*	11	8	0.630
Good response to chemotherapy, *n*	6	4	0.697

*Note:* No significant differences were observed between the two groups with independent sample *t*‐test (age) and Fisher's exact test (sex, metastasis, chemotherapeutic response). The overall survival (OS) of the mTKI and the control group was compared through survival analysis. OS was evaluated from the time of progression or relapse after second‐line treatment to the time of death. The log‐rank test revealed a significant difference in the survival distribution between patients receiving mTKIs and those who did not (chi2(1) = 8.13 *p* = 0.004). The Kaplan–Meier curve is illustrated in Figure 2.

According to the Cox regression analysis, there is a statistically significant difference (*p* = 0.001) in the hazards for death between OSC patients receiving mTKI treatment and those in the control group. Patients under mTKI treatment exhibit a lower hazard compared to the control group, with a hazard ratio of 0.14 (95% CI: 0.04–0.43). This indicates a substantial decrease in the risk of death among mTKI patients. Importantly, these results remain robust after adjusting for potential confounding factors such as age, metastatic disease, and response to chemotherapy. Detailed results are in Table 3.

**TABLE 3:** Summary statistics of the survival analysis by group.

	Time at risk (follow up time, months)	Incidence rate	*N*	25% survival time (months)	50% survival time (months)	75% survival time (months)
Control	48.17	0.23	11	0.67	1.07	4.43
mTKI	174.3	0.07	13	4.37	13.47	25.5
Total	222.47	0.1	24	1.07	3.37	14.27

**FIGURE 3:**
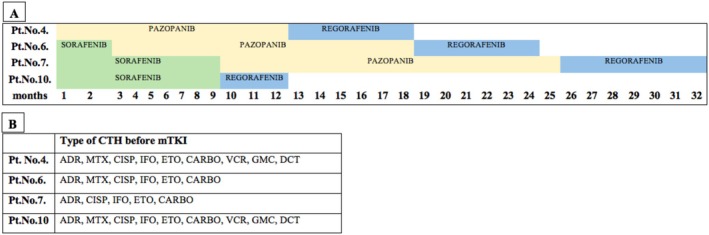
(Panel A) shows the timeline of the patients who were treated with multiple types of mTKIs. (Panel B) shows the types of chemotherapies given prior to the initiation of mTKI treatment. ADR, Adriamycin; CARBO, Carboplatin; CISP, Cisplatin; CTH, Chemotherapy; DCT, Docetaxel; ETO, Etoposide; GMC, Gemcitabine; IFO, Ifosfamide; MTX, Methotrexate; VCR, Vincristine.

A total of 4 patients received multiple types of mTKIs. The treatment timeline is shown in Figure 3. A total of 2 patients received 3, while the other 2 received 2 types of mTKIs for several months. The decision to switch to a new agent was based on the progression of the disease as confirmed by radiological examination.

### 3.4 Outcomes for Chondrosarcoma, Rhabdomyosarcoma and Synovial Sarcoma Patients

One patient with mesenchymal CSC received pazopanib following the first relapse. Diagnosed with CSC in the proximal region of the left femur at the age of 13.8, the patient presented with multiple pulmonary metastases detected during the initial chest CT examination. While surgical removal of the primary tumor was performed, achieving an R0 resection was not feasible. Subsequent to chemotherapy with methotrexate, adriamycin, ifosfamide, carboplatin, and etoposide, the primary tumor site was irradiated, and the pulmonary metastases were excised. A pulmonary relapse occurred 25 months post‐diagnosis, leading to the initiation of pazopanib after lesion removal. The patient underwent 6 months of mTKI therapy, achieving stable disease. However, 5 months post‐mTKI therapy, the patient experienced a 2nd relapse. Despite undergoing surgeries, chemotherapy, and pembrolizumab therapy, the patient had two additional relapses and died of the disease.

Another patient with embryonal RMS of the left lower limb received regorafenib. Chemotherapy was started following partial tumor removal, but showed progression during the treatment course. Second‐line chemotherapy was initiated and limb amputation was performed. 3 months post completing the second‐line therapy, the patient relapsed. Palliative irradiation and regorafenib were initiated. Following 5 months of mTKI treatment, the patient passed away due to disease progression.

In the case of 3 SySa patients, pazopanib treatment turned out to be especially effective. In all 3 cases, the disease showed great sensitivity to pazopanib. In the first case, first‐ and second‐line chemotherapy was ineffective. After 5 months of pazopanib therapy, the tumor showed 20% regression, and operation was successfully performed; CR was achieved. Two patients had relapsed disease, and after first‐ and second‐line treatment, pazopanib was initiated, and PR was achieved. To date, both patients are alive with residual disease. Table 4 shows the details of the patients' case history.

**TABLE 4:** Characteristics of synovial sarcoma patients undergoing mTKI therapy.

Patient No.	Age at diagnosis (years)	Localisation	Chemotherapies	Indication for mTKI	Best response with mTKI treatment	Status at first MRI after initiation of mTKI	Length of therapy (months)	Current status
Pt. 16.	16.3	Right inguinal region (canalis femoralis)	IFO, VCR, ADR	Progression during first line chemotherapy	CR	20% regression	11	NED
Pt. 17.	9	Left lower limb	IFO, VCR, ADR, Act‐D, TOP, CARBO, CYC	Inadequate response to 1st and 2nd line therapy	PR	45% regression	9 (under therapy)	AWD
Pt. 18.	9.4	Left cubital region	IFO, VCR, ADR, Act‐D, TOP, CARBO, CYC	2nd relapse	PR	Regression of the postoperative lesions	7 (under therapy)	AWD

Abbreviations: Act‐D, actinomycin‐D; ADR, adriamycin; AWD, alive with disease; CARBO, carboplatin; CYC, cyclophosphamide; IFO, ifosfamide; NED, no evidence of disease; TOP, topotecan; VCR, vincristine.

## 4 Adverse Effects and Toxicities

A total of 11 out of the 18 patients experienced at least one type of adverse effect (AE) during mTKI treatment, with seven patients encountering multiple AEs. The most common AE during pazopanib and sorafenib treatment was hair hypopigmentation, reported in 10 cases. Diarrhea occurred in nine cases, typically managed with loperamide. Skin rashes or erythematous lesions were noted in four cases, with one case necessitating surgical intervention due to severe anal fissures and bleeding. Furthermore, hypertension and gynaecomastia were reported in one case each. In four cases, dose adjustments or suspension of therapy were necessary due to gastrointestinal or skin symptoms.

## 5 Quality of Life

The patients showed a generally good performance status based on the Karnofsky‐Lansky scores throughout the therapy. Those with lower scores typically experienced rapid progression despite mTKI administration. Overall, it can be concluded that the treatment was well tolerated and easily incorporated into daily life. The therapy did not require prolonged hospital stays, as follow‐up examinations were conducted on an outpatient basis.

## 6 Discussion

The poor prognosis of relapsed or progressive bone and soft tissue tumors shows the importance of the development of new therapeutic strategies.

There is an increasing awareness regarding the biology of specific kinases and signaling pathway activations in bone tumor cells [13, 14]. By comparing the targets of mTKIs, studies indicate that VEGFRs and RET are the key targets for osteosarcoma treatment. Furthermore, a review of the literature suggests that KIT, PDGFRs, MET, IGF‐1R, and AXL could also serve as potential therapeutic targets for osteosarcoma. However, clinical trials in osteosarcoma have shown that single‐target therapy, such as inhibiting VEGFs (with bevacizumab), KIT and PDGFRα (with imatinib), or IGF‐1R (with cixutumumab or R1507) has limited efficacy. These findings suggest that targeting a single receptor is not a viable approach for osteosarcoma treatment. This is likely due to significant crossover and overlap among the downstream signaling pathways of receptor tyrosine kinases [15]. In addition, due to the inhibition of multiple targets, the likelihood of developing resistance to therapy might decrease.

There are various types of mTKIs under investigation and used off‐label. In clinical trials, mTKIs showed activity in a wide range of malignancies in adults [9] and children [16, 17]. In randomized, placebo‐controlled evaluations, mTKIs as monotherapy in refractory bone tumors showed the prolongation of 4‐month progression‐free survival (PFS). In these settings, 4‐month PFS for the mTKI group was 65% and 44% in comparison with 0% and 10% in the placebo group [18, 19].

In our retrospective cohort study, we evaluated mTKI treatment in pediatric sarcomas in terms of tolerability and survival. The majority of our patients treated with mTKIs had relapsed, therapy‐resistant, or progressive OSC. Following first and second‐line chemotherapy, along with surgical treatment, mTKIs emerged as the sole therapeutic option for these patients as a palliative measure. The objective of the therapy is to slow progression and sustain a good quality of life. In our cohort, the median duration of therapy was 10 months, with a median time to progression of 4 months. Notably, 5 cases exhibited an extended duration of mTKI treatment, with 4 receiving multiple types of mTKIs. This suggests potential benefits of initiating new agents even in slowly progressing diseases, potentially extending patients' lives by months.

We conducted a comparison of the survival outcomes of OSC patients receiving mTKIs against a control group. Overall survival was analyzed from the point of progression or relapse after the second‐line treatment. Our findings revealed significantly reduced hazards of death among the mTKI patients compared to the control group. Even though mTKIs are not considered curative treatments, they show promise in slowing disease progression.

The management of relapsed or therapy‐resistant SySa might be challenging. In addition to recurrent surgical interventions, therapeutic options may include high‐dose continuous ifosfamide infusion, trabectedin, and pazopanib. Of these, pazopanib was the first anti‐angiogenic drug approved for soft tissue sarcomas. Recent analysis of the clinical trials revealed that 36% of STS patients treated with pazopanib achieved a progression free survival (PFS) exceeding 6 months, while 34% survived beyond 18 months, and 3.5% remained progression‐free for over 2 years [20]. Recently, a meta‐analysis of 7 studies focusing on SySa reported a median PFS of 5.3 months and an overall response rate of 18.9% for patients treated with pazopanib [21]. Based on the outcomes of the aforementioned studies, the benefits of pazopanib treatment in most STS patients appear modest; however, a small subset of patients exhibits long‐term positive responses. In our study, we observed sustained benefits from mTKI treatment in 3 SySa patients, with pazopanib demonstrating high effectiveness and no progression observed thus far.

Regarding the side effects, the most frequently observed ones during treatment were diarrhea and hair hypopigmentation, the latter occurring with sorafenib and pazopanib therapy.

Based on studies conducted primarily in the adult population, in addition to diarrhea, rash‐like skin symptoms and hand‐foot‐mouth reactions are also particularly common with the use of these drugs [9, 22]. Other common side effects include hypertension, fatigue, loss of appetite, and nausea; however, these symptoms were not frequently observed in the patient group we studied. If these symptoms worsen, the patient may require hospitalization due to the need for parenteral fluid replacement or surgical interventions. Since it is crucial in palliative care to maximize the patient's comfort and time outside the hospital, it is essential to carefully weigh the cost–benefit ratio during treatment.

The limitations of our study include the small, heterogeneous patient cohorts and the retrospective nature of the data. The comparison of OSC patients receiving mTKIs to controls was not randomized. Despite both groups following identical chemotherapeutic treatment schedules, certain variables, such as time from initial symptoms to diagnosis and the type and timing of surgical interventions, especially regarding the surgical resections of pulmonary metastases, were not accounted for, potentially introducing bias to the results. Additionally, further limitations of the study include the lack of a direct comparison of the efficacy and tolerability of the three drugs, as well as the small sample size, which required us to evaluate patients receiving different types of mTKIs as a single group. Since this is a single‐centre study, the applicability to a larger population remains uncertain. Furthermore, our study cannot provide insight into the long‐term adverse effects of the aforementioned drugs, given the short follow‐up period.

Nevertheless, our finding suggests that mTKIs are well‐tolerated and can serve as a therapeutic option for metastatic, refractory bone sarcomas as palliative treatment, aiming to slow disease progression and uphold a good quality of life. Pazopanib usage should be considered as a second‐ or third‐line treatment in SySa, supported by favorable outcomes from numerous trials and case studies. Further investigations are essential to delineate the optimal utilization of mTKIs in the management of this group of pediatric patients.

## Author Contributions

**Anna Mohás:** writing – original draft (equal). **Klára Horváth:** writing – original draft (equal). **Monika Csóka:** writing – original draft (equal). **Zsuzsanna Jakab:** data curation (equal), supervision (equal).

## Conflicts of Interest

The authors declare no conflicts of interest.

## Data Availability

The data are not publicly available due to privacy or ethical restrictions.

## References

[1] S. Smeland , S. S. Bielack , J. Whelan , et al., “Survival and Prognosis With Osteosarcoma: Outcomes in More Than 2000 Patients in the EURAMOS‐1 (European and American Osteosarcoma Study) Cohort,” European Journal of Cancer 109 (2019): 36–50, 10.1016/j.ejca.2018.11.027. 30685685 PMC6506906

[2] S. X. Skapek , A. Ferrari , A. A. Gupta , et al., “Rhabdomyosarcoma,” Nature Reviews Disease Primers 5, no. 1 (2019): 1. 10.1038/s41572-018-0051-2 PMC7456566 30617281

[3] A. Ferrari , I. Sultan , S. L. Spunt , et al., “Soft Tissue Sarcoma Across the Age Spectrum: A Population‐Based Study From the Surveillance Epidemiology and End Results Database,” Pediatric Blood & Cancer 57 (2011): 943–949. 21793180 10.1002/pbc.23252 PMC4261144

[4] J. Coventon , “A Review of the Mechanism of Action and Clinical Applications of Sorafenib in Advanced Osteosarcoma,” Journal of Bone Oncology 8 (2017): 4–7. 28828294 10.1016/j.jbo.2017.07.001 PMC5552021

[5] G. Grignani , E. Palmerini , P. Dileo , et al., “A Phase II Trial of Sorafenib in Relapsed and Unresectable High‐Grade Osteosarcoma After Failure of Standard Multimodal Therapy: An Italian Sarcoma Group Study,” Annals of Oncology 23, no. 2 (2012): 508–516, 10.1093/annonc/mdr151. 21527590

[6] G. Grignani , E. Palmerini , V. Ferraresi , et al., “Sorafenib and Everolimus for Patients With Unresectable High‐Grade Osteosarcoma Progressing After Standard Treatment: A Non‐Randomised Phase 2 Clinical Trial,” Lancet Oncology 16, no. 1 (2015): 98–107. 25498219 10.1016/S1470-2045(14)71136-2

[7] A. Grothey , E. Van Cutsem , A. Sobrero , et al., “Regorafenib Monotherapy for Previously Treated Metastatic Colorectal Cancer (Correct): An International, Multicentre, Randomised, Placebocontrolled, Phase 3 Trial,” Lancet 381 (2013): 303–312. 23177514 10.1016/S0140-6736(12)61900-X

[8] L. E. Davis , V. Bolejack , R. G. Maki , et al., “Randomized Double‐Blind Phase II Study of Regorafenib in Patients With Metastatic Osteosarcoma,” Journal of Clinical Oncology 37, no. 16 (2019): 1424–1431. 31013172 10.1200/JCO.18.02374 PMC7799443

[9] W. T. van der Graaf , J. Y. Blay , S. P. Chawla , et al., “Pazopanib for Metastatic Soft‐Tissue Sarcoma (PALETTE): A Randomised, Double‐Blind, Placebo‐Controlled Phase 3 Trial,” Lancet 379 (2012): 1879–1886. 22595799 10.1016/S0140-6736(12)60651-5

[10] S. S. Bielack , B. Kempf‐Bielack , G. Delling , et al., “Prognostic Factors in High‐Grade Osteosarcoma of the Extremities or Trunk: An Analysis of 1,702 Pattreated on Neoadjuvant Cooperative Osteosarcoma Study Group Protocols,” Journal of Clinical Oncology 20, no. 3 (2002): 776–790. 11821461 10.1200/JCO.2002.20.3.776

[11] M. Sparber‐Sauer , A. Ferrari , D. Kosztyla , et al., “Long‐Term Results From the Multicentric European Randomized Phase 3 Trial CWS/RMS‐96 for Localized High‐Risk Soft Tissue Sarcoma in Children, Adolescents, and Young Adults,” Pediatric Blood & Cancer 69, no. 9 (2022): e29691. 35441463 10.1002/pbc.29691

[12] M. L. Joseph , A. M. Di Bisceglie , J. Bruixa , et al., “For the Panel of Experts in HCC‐Design Clinical Trials, Design and Endpoints of Clinical Trials in Hepatocellular Carcinoma,” JNCI Journal of the National Cancer Institute 100, no. 10 (2008): 698–711, 10.1093/jnci/djn. 18477802

[13] J. McGuire , T. J. Utset‐Ward , D. R. Reed , and C. C. Lynch , “Re‐Calculating! Navigating Through the Osteosarcoma Treatment Roadblock,” Pharmacological Research 117 (2017): 54e64. 27940205 10.1016/j.phrs.2016.12.004

[14] W. Jin , “The Role of Tyrosine Kinases as a Critical Prognostic Parameter and Its Targeted Therapies in Ewing Sarcoma,” Frontiers in Cell and Developmental Biology 8 (2020): 613. 32754598 10.3389/fcell.2020.00613 PMC7381324

[15] Z. Tian , X. Niu , and W. Yao , “Receptor Tyrosine Kinases in Osteosarcoma Treatment: Which Is the Key Target?,” Frontiers in Oncology 10 (2020): 1642, 10.3389/fonc.2020.01642. 32984034 PMC7485562

[16] A. R. Weiss , Y.‐L. Chen , T. J. Scharschmidt , et al., “Pathological Response in Children and Adults With Large Unresected Intermediate‐Grade or High‐Grade Soft Tissue Sarcoma Receiving Preoperative Chemoradiotherapy With or Without Pazopanib (ARST1321): A Multicentre, Randomised, Open‐Label, Phase 2 Trial,” Lancet Oncology 21 (2020): 1110–1122. 32702309 10.1016/S1470-2045(20)30325-9 PMC7745646

[17] A. D. J. Pearson , N. Gaspar , K. Janeway , et al., “Paediatric Strategy Forum for Medicinal Product Development of Multi‐Targeted Kinase Inhibitors in Bone Sarcomas ACCELERATE in Collaboration With the European Medicines Agency With Participation of the Food and Drug Administration,” European Journal of Cancer 173 (2022): 71–90. 35863108 10.1016/j.ejca.2022.06.008

[18] F. Duffaud , O. Mir , P. Boudou‐Rouquette , et al., “Efficacy and Safety of Regorafenib in Adult Patients With Metastatic Osteosarcoma: A Non‐Comparative, Randomised, Double‐Blind, Placebo‐Controlled, Phase 2 Study,” Lancet Oncology 20 (2019): 120–133. 30477937 10.1016/S1470-2045(18)30742-3

[19] A. Italiano , O. Mir , S. Mathoulin‐Pelissier , et al., “Cabozantinib in Patients With Advanced Ewing Sarcoma or Osteosarcoma (CABONE): A Multicentre, Single‐Arm, Phase 2 Trial,” Lancet Oncology 21 (2020): 446e55. 32078813 10.1016/S1470-2045(19)30825-3 PMC8763616

[20] B. Kasper , S. Sleijfer , S. Litière , et al., “Long‐Term Responders and Survivors on Pazopanib for Advanced Soft Tissue Sarcomas: Subanalysis of Two European Organisation for Research and Treatment of Cancer (EORTC) Clinical Trials 62043 and 62072,” Annals of Oncology 25, no. 3 (2014): 719–724. 24504442 10.1093/annonc/mdt586 PMC4433518

[21] C. Carroll , N. Patel , N. B. Gunsoy , H. A. Stirnadel‐Farrant , and S. Pokras , “Meta‐Analysis of Pazopanib and Trabectedin Effectiveness in Previously Treated Metastatic Synovial Sarcoma (Second‐Line Setting and Beyond),” Future Oncology 18 (2022): 3651–3665. 36399116 10.2217/fon-2022-0348

[22] J. Bruix , S. Qin , P. Merle , et al., “Regorafenib for Patients With Hepatocellular Carcinoma Who Progressed on Sorafenib Treatment (RESORCE): A Randomised, Double‐Blind, Placebo‐Controlled, Phase 3 Trial,” Lancet 389 (2017): 56–66, 10.1016/S0140-6736(16)32453-9. 27932229

